# Development and Psychometric Evaluation of the Nursing Instructors’ Clinical Teaching Performance Inventory

**DOI:** 10.5539/gjhs.v7n3p30

**Published:** 2014-10-28

**Authors:** Mansoureh A. Farahani, Hormat Sadat Emamzadeh Ghasemi, Nasrin Nikpeyma, Zhila Fereidouni, Maryam Rassouli

**Affiliations:** 1 School of Nursing and Midwifery, Iran University of Medical Sciences, Tehran, Iran; 2 Nursing Management Departments, Schools of Nursing and Midwifery, Tehran University of Medical Sciences, Tehran, Iran; 3 Community Health Departments, School of Nursing and Midwifery, Tehran University of Medical Sciences. Tehran, Iran; 4 School of Nursing, Fasa University of Medical Sciences, Fasa, Fars, Iran; 5 Paediatric Nursing Departments, School of Nursing & Midwifery, Shahid Beheshti University of Medical Sciences, Tehran, Iran

**Keywords:** psychometric evaluation, nursing instructors, teaching performance evaluation, clinical education, methodological research

## Abstract

Evaluation of nursing instructors’ clinical teaching performance is a prerequisite to the quality assurance of nursing education. One of the most common procedures for this purpose is using student evaluations. This study was to develop and evaluate the psychometric properties of Nursing Instructors’ Clinical Teaching Performance Inventory (NICTPI). The primary items of the inventory were generated by reviewing the published literature and the existing questionnaires as well as consulting with the members of the Faculties Evaluation Committee of the study setting. Psychometric properties were assessed by calculating its content validity ratio and index, and test-retest correlation coefficient as well as conducting an exploratory factor analysis and an internal consistency assessment. The content validity ratios and indices of the items were respectively higher than 0.85 and 0.79. The final version of the inventory consisted of 25 items, and in the exploratory factor analysis, items were loaded on three factors which jointly accounting for 72.85% of the total variance. The test-retest correlation coefficient and the Cronbach’s alpha of the inventory were 0.93 and 0.973, respectively. The results revealed that the developed inventory is an appropriate, valid, and reliable instrument for evaluating nursing instructors’ clinical teaching performance.

## 1. Introduction

Clinical education is a key component of nursing education (Thornton & Chapman, 2000). In clinical education, considerable learning opportunities are provided to students to develop and enhance their professional skills and qualify them for actual nursing practice (Thornton & Chapman, 2000; Watson, Stimpson, Topping, & Porock, 2002). Nursing instructors have an influential role in providing quality clinical education. It is believed that the quality of nursing care is directly related to clinical instructors’ level of competence (Fluit, Bolhuis, Grol, Laan, & Wensing, 2010).

Instructors’ competence and proficiency are largely dependent on the accurate performance evaluations that focus on improving their performance (Emamzadeh Ghasemi, Rafii, Farahani, & Mohammadi, 2014). Heshmati-Nabavi and Vanaki (2009) reported that incongruence between evaluation criteria and instructors’ competencies negatively affects their effectiveness and efficiency. Accordingly, accurate evaluation of instructors’ performance is a stepping stone in having competent nursing instructors and graduates (Heshmati-Nabavi & Vanaki, 2010; Reeve, 1994).

Generally, evaluation is referred to the systematic process of data collection, analysis, and interpretation for identifying one’s success in attaining predefined goals. During performance evaluation, extensive data are gathered for judging instructors’ competence in teaching, improving their clinical teaching performance, and promoting students’ learning (K. D. Peterson & C. A. Peterson, 2006). The results of performance evaluation provide employees with useful information about employers’ expectations and help them identify their own strengths and weaknesses (Marquis & Huston, 2009). Accordingly, the main aim of evaluation is quality improvement (Morrison, 2003; Salsali, 2005; Ziaee, Miri, Haji-abadi, Azarkar, & Eshbak, 2007). Scriven et al. (2005) also noted that performance evaluation greatly enhances the quality of services provided by an organization.

An essential prerequisite to accurate evaluations is the availability of reliable and valid assessment tools. Reliable and valid tools provide accurate and credible information, are user-friendly, provide the opportunity for giving immediate feedback, and help users attain the goals of evaluation (Houser, 2008). Adhami et al. (2000) also showed that the positive effects of evaluation are brought about only by using reliable and valid tools. Consequently, prior to performing any educational evaluation, it is necessary to define the criteria of educational achievement as well as the indicators for instructors’ success in fulfilling such criteria (Kashaninia, Rasuli, Hoseini, & Kashef Ghorbanpor, 2013; Reeve, 1994).

One of the most common procedures for evaluating instructors’ clinical teaching performance is using student ratings (Beran & Rokosh, 2009; Howard, 1998; Shakournia, Elhampour, Mozafari, & DashtBozorgi, 2008). Educational administrators, both formally and informally, use students’ opinions as a criterion for judging instructors’ competence (Seldin, 1993). In a review study, Wachtel (1998) found that student evaluation of college and university instructors’ teaching performance is a valid indicator for evaluating the effectiveness of teaching. Student evaluations are widely used worldwide as an effective and reliable method for teaching evaluation. However, the validity of such evaluations has been almost questioned as a result of lack in valid and reliable assessment tools (Shakournia et al., 2008). Pazargadi et al. (2008) also reported that lack of the valid and reliable assessment tools as a major barrier to evaluation of nursing instructors’ teaching performance. Accordingly, we conducted this study to bridge this gap. So, the aim of this study was to develop and evaluate the psychometric properties of the Nursing Instructors’ Clinical Teaching Performance Inventory (NICTPI).

## 2. Method

This was a methodological study conducted in 2011. The aim of methodological studies is the development and psychometric evaluation of data collection tools (LoBiondo-Wood & Haber, 2013). The study population consisted of nursing instructors and students of in school of nursing and midwifery of Tehran University of Medical Sciences. This study was carried out in two successive phases that namely, Phase 1: Item generation phase, Phase 2: Psychometric evaluation phase.

In the first phase, we generated the primary items by reviewing the published literature as well as the questionnaires on nursing instructors’ teaching performance retrieved from the Education Development Organization of the study setting. Then, we invited the members of the Faculties Evaluation Committee of the study setting to four focus group interviews and a two-round Delphi exploration to generate the final item pool. Finally, an item pool of 27 items was generated.

The Second phase, Psychometric evaluation of NICTPI was conducted through assessing its face, content, and constructs validity as well as its internal consistency and test-retest stability.

### 2.1 Content Validity Assessment

Content Validity Ratio (CVR) and the Content Validity Index (CVI) were calculated for assessing the content validity of NICTPI. For calculating CVR, we invited thirteen nursing instructors who were experienced in the fields of teacher evaluation and instrument development. Accordingly, we provided them with the item pool and asked them to rate each item on a three-point scale on which 1 stood for ‘Essential’, 2 for ‘Useful but not essential’, and 3 for ‘unessential’. Then, we used panelists’ ratings and the CVR formula for calculating the CVR of each item. Finally, given the number of panelists, items with a CVR of 0.54 or higher were selected.

For calculating the CVI of NICTPI, we invited ten faculties affiliated to the Nursing Management and Medical-Surgical Nursing Departments of the study setting. Panelists were asked to determine the ‘relevancy’, ‘clarity’, and ‘simplicity’ of the items on four-point scale as recommended by Waltz and Bausell (1983).

### 2.2 Face Validity Assessment

We strived to use a clear and appropriate wording for the items. Moreover, we asked twelve medical-surgical nursing students to read and evaluate the wording of the items. We also used the panelist’ comments in the content validity assessment phase for improving the face validity of the inventory. Some minor changes were made according to students and panelists’ comments.

### 2.3 Construct Validity Assessment

The construct validity of NICTPI was assessed by using the exploratory factor analysis method. Polit and Beck (2012) recommended that a sample of 3–10 people per item is needed for an exploratory factor analysis. Accordingly, we recruited a random sample of 175 nursing students who were passing their clinical courses. All the students were recruited from the study setting by using the stratified and the simple random sampling methods. Students’ year of education at university was considered as sampling strata. Accordingly, we used the simple random sampling method for recruiting proportionate number of students from each stratum. Thirty eight questionnaires were filled incompletely which were accordingly excluded from the analysis. Finally, 137 questionnaires were included in factor analysis.

Two main tests, Kaiser–Meyer–Olkin (KMO) and Bartlett’s Test for Sphericity, were used to measure of sampling adequacy and estimate the appropriateness of data. Also, the varimax rotation was used for making factor interpretation easier.

### 2.4 Reliability Assessment

We assessed the reliability of NICTPI by using the test-retest and the internal consistency methods. Accordingly, 20 students were asked to evaluate their clinical instructor by using the final version of the inventory. Their responses were used for calculating its Coronbach’s alpha. Ten days later, the same sample of students was asked to respond to the inventory for the second time. The two sets of responses were used for assessing the test-retest reliability of the inventory. All data analyses were done by using the statistical package for social sciences (SPSS, v. 16.0).

## 3. Ethical Consideration

This study was approved by the institutional review board of the nursing research center of Iran University of Medical Sciences. We ensured the study participants that their provided data would be managed and reported confidentially and anonymously.

## 4. Findings

Most of the study participants (70.07%) were female. The mean of participants’ age was 22.50±2.80.

### 4.1 Content Validity

According to Lawshe (1975), the minimum acceptable value of CVR for thirteen panelists is 0.54. In our study, the CVRs of all items were higher than 0.54 and hence, no item was removed. Moreover, items that had a CVI of higher than 0.79 remained in the final version of the inventory. Consequently, two items which had a CVI of less than 0.79 were removed from the inventory. These items were ‘Your general evaluation of this clinical course’ and ‘your regular attendance at this course’. Consequently, at the end of the content validity assessment phase, 25 items remained in the inventory.

### 4.2 Construct Validity

The results of the Bartlett’s test of sphericity confirmed the appropriateness of the factor analysis (χ^2^=5395.271 and P value < 0.001). Moreover, the Kaiser-Meyer-Olkin was equal to 0.954, showing sampling adequacy. The varimax rotation was used for making factor interpretation easier. In addition, eigenvalues greater than 1 were used for determining of main factors. Items that had a factor load of greater than 0.3 were considered as acceptable. Accordingly, the results of the exploratory factor analysis with varimax rotation showed a three-factor structure for the 25-item NICTPI with a total variance of 72.85%. These three factors were ‘Orienting students to the rules and regulations of the course’, ‘the process of clinical teaching’, and ‘Instructor’s professional knowledge, attitude, and competence’ (Table 1). None of the items were removed from the inventory. Consequently, the final version of NICTPI consists of 25 items scored on five-point likert scale range, from 1 (Poor) to 5 (Very good).

**Table 1:** Summary of principal components (with eigenvalues) contributing the items as a result of explorative factor analysis of the NICTPI

Items	Factor Loading^+^		

	Factor 1	Factor 2	Factor 3
1. Orienting students to the physical environment of the ward, personnel, and the types of diseases managed in the ward	0.407		0.686
2. Orienting students to the aims and the objectives of the course as well as their duties and assignments at the beginning of the course	0.555		0.702
3. Orienting students to the evaluation criteria at the beginning of the course	0.411		0.706
4. Effective planning and supervision for students’ timely arrival and regular attendance at ward		0.783
5. Formative evaluation of students’ learning by using predetermined criteria	0.436	0.471	0.578
6. Providing students with regular constructive feedbacks to improve their abilities and overcome their weaknesses		0.582	0.657
7. Daily planning for attaining the goals of clinical education		0.584	0.575
8. Head-nurse or ward authorities’ awareness of students’ daily work schedule		0.305
9. Guiding students in providing nursing care to patients hospitalized in the ward		0.457	0.710
10. Guiding students in providing patient education based on patients’ educational needs		0.544	0.700
11. Supervising students or having a staff nurse or a senior student supervise them during implementing special procedures	0.499	0.420
12. Training nursing procedures that are specific to current clinical course		0.623	0.457
13. Planning for visiting other settings that are related to the current clinical course		0.679
14. Providing clinical educations based on the nursing process		0.644	0.440
15. Providing clinical educations based on patients’ educational needs		0.607	0.600
16. Teaching students about how to write daily clinical reports and nursing notes based on the nursing process		0.836
17. Receiving or having a staff nurse receive students’ handoff reports at the end of each working shift	0.600	0.547
18. Teaching students about how to provide care before and after diagnostic procedures		0.564	0.563
19. Informing students about evaluation criteria and their final score up to one week after the completion of the course	0.413	0.443	0.432
20. Taking ethical considerations into account when dealing with patients, students, and hospital personnel			0.724
21. Creating a teaching-learning environment that welcomes criticism and recommendations		0.446	0.678
22. Having interest in nursing			0.796
23. Having interest in nursing education		0.428	0.745
24. Having the ability to be a good role-model for students		0.531	0.710
25. Having the ability to encourage and motivate students for providing care to patients		0.630	0.568

All factor loadings>0.30 are reported. ^+^After Varimax rotation. Factor 1: Orienting students to the rules and regulations of the course; Factor 2: The process of clinical teaching; Factor 3: Instructor’s professional knowledge, attitude, and competence.

### 4.3 Reliability

The results of the internal consistency assessment revealed a Coronbach’s alpha of 0.973 for NICTPI. Moreover, the Pearson product moment correlation test revealed that the test-retest correlation coefficient was equal to 0.93. These findings confirmed the stability and the internal consistency of the inventory.

## 5. Discussion

Evaluation of nursing instructors’ clinical teaching performance is performed differently worldwide. One of the most commonly used methods is student evaluation of instructors’ performance (Beran & Rokosh, 2009; Seldin, 1993). An important factor affecting the accuracy and the precision of these evaluations is the use of simple, valid, reliable, and interpretable instruments (Howard, 1998; Shakournia et al., 2008). According to Houser (2008), valid and reliable instruments measure the intended quality with sufficient accuracy and produce consistent results at repeated measurements.

The findings of the study revealed that this inventory had satisfactory face, content, and construct validity as well as an acceptable internal consistency and test-retest stability. The construct validity of NICTPI was assessed by conducting an exploratory factor analysis which revealed that the inventory consisted of three dimensions. The dimensions were ‘Orienting students to the rules and regulations of the course’, ‘the process of clinical teaching’, and ‘Instructor’s professional knowledge, attitude, and competence’. Sand-Jekin (2006) also developed a form for evaluating clinical instructors’ performance was consisted of fourteen dimensions. Some dimensions of the form were orienting students to clinical environment, informing them about expectations and objectives, being accessible to them, creating of an ideal learning environment, having professional competence, and giving students constructive feedbacks (Sand-Jecklin, 1996). It is evident that there are great similarities between the evaluation form developed by Sand-Jeklin (1996) and NICTPI. However, we strived to develop a more specific context-based instrument which is compatible with the culture and the policies of the Iranian nursing education system.

Our findings revealed that factors such as paying attention to students’ educational needs, orienting them to the rules and regulations of clinical courses, effective management of clinical teaching-learning process, and instructor’s professional knowledge, attitude, and competence were among the most important factors in the evaluation of nursing clinical instructors. Amini et al. (2012) reported that clinical instructors’ role-modeling roles such as demonstration of professional competency, clear commitment to moral principles, and effective supervision of students’ learning are the main components in evaluating their clinical teaching performance. Heshmati-Nabavi and Vanaki (2010) also reported that the main attributes of an effective clinical instructor include having great professional and clinical competencies, having the ability to transfer knowledge to practice, maintaining unity between words and deeds, and creating a supportive and enjoyable learning environment. Young (2009) also developed a 22-item likert-type instrument for student evaluation of clinical instructors. The items of this instrument included, but not limited to, transferring knowledge to practice, emphasizing professional responsibilities, respecting students’ beliefs, treating and evaluating students with fairness, providing constructive feedbacks, and attending regularly at clinical learning environment for guiding and supervising students. Although several items of the Young’s instrument are similar NICTPI, the former is comparatively briefer.

The high Cronbach’s value of NICTPI demonstrates its great internal consistency and acceptable reliability. The reliability of NICTPI was also confirmed by using the test-retest stability assessment method. Given the high Cronbach’s alpha and great internal consistency of the inventory, the possibility of item reduction can be examined in prospective studies to generate its short form versions.

Despite the questionable validity and reliability of student evaluations of instructors’ performance, many universities around the world consider students as a valuable source of information for clinical education quality assurance. To minimize students’ biases toward evaluations of instructors’ teaching performance, Marofi et al. (2007) recommended the involvement of instructors in the development of evaluation forms. In the current study, we strived to involve numerous nursing faculties and clinical instructors in the process of development and psychometric evaluation of NICTPI.

## 6. Conclusion

NICTPI is an appropriate instrument for evaluating nursing instructors’ clinical teaching performance with an acceptable validity and reliability. Despite its relative briefness, NICTPI covers the key aspects of clinical instructors’ teaching performance evaluation. Moreover, because of its likert type, NICTPI is an easy-to-use time-saving instrument. However, given the complexity and multidimensionality of clinical teaching, we strongly recommend using other evaluation procedures—besides NICTPI—for obtaining more accurate and more reliable results. These procedures may include self evaluation, peer evaluation, and evaluation by managers.
